# Psychological dimensions in alcohol use disorder: comparing active drinkers and abstinent patients

**DOI:** 10.3389/fpsyt.2024.1420508

**Published:** 2024-06-27

**Authors:** Alessio Zizzi, Isabel Margherita Berri, Alessandro Berri, Martina Occhipinti, Andrea Escelsior, Riccardo Guglielmo, Beatriz Pereira Da Silva, Mario Amore, Gianluca Serafini

**Affiliations:** ^1^ Department of Neuroscience, Rehabilitation, Ophthalmology, Genetics, and Mother-Child, School of Medical and Pharmaceutical Sciences, University of Genoa, Genova, Italy; ^2^ University of Genoa, Genoa, Italy; ^3^ San Martino Hospital (IRCCS), Genova, Italy

**Keywords:** alcohol use disorder, suicide risk, abstinence, impulsivity, alexithymia

## Abstract

**Background:**

Alcohol use disorder (AUD) is a major public health concern due to its various physical, psychological, and social consequences. Despite regulatory differences, abstinence remains the primary treatment objective. Addressing the multifaceted nature of alcohol use disorder requires a comprehensive approach.

**Methods:**

150 AUD patients (66%male) with a mean age of 54.10 ± 11.3 years were recruited for the study. Depression, impulsivity, alexithymia, and hopelessness were assessed to determine if there were significant differences in these dimensions between abstinent (N=72) and active drinkers (N=78).

**Results:**

The study found significant differences in the psychological dimensions scores, active drinkers exhibited higher levels of depression, impulsivity, alexithymia, and hopelessness compared to abstinent patients.

**Conclusion:**

Treatment outcomes for patients with AUD vary between regulatory agencies, but abstinence remains the safest and most preferred objective in managing AUD. Prioritizing abstinence-oriented interventions is crucial for achieving long term recovery and minimizing relapse risk. These results emphasize the intricate relationship between AUD and mental health issues, highlighting the need for comprehensive interventions addressing both alcohol consumption and associated psychological distress. Promoting abstinence (or at least reducing alcohol consumption) not only preserves mental health but also prevents life-threatening consequences such as suicide.

## 1 Introduction

### 1.1 Alcohol use disorder

Alcohol use disorder (AUD) is an insidious disorder, characterized by an often intermittently relapsing course. In Europe, AUD affects approximately 66.2 million people aged 15 and over, predominantly men, with Italy showing one of the lowest prevalences, both for men (1.7%) and women (1.0%) (1). As an individual relapses into drinking, they become more likely to develop a variety of additional somatic complications. including metabolic disorders, hypertension and cerebrovascular diseases (2). The World Health Organization (WHO), ranks harmful alcohol consumption as the third risk factor for premature death and disability in the world (3).The consequences related to AUD do not only concern the physical sphere, but also extend to psychological and social spheres (4–6).

AUD increases the risk of physical harm and dangerous behavior and impacts school and work performance, childcare and household responsibilities, among other things. Using alcohol in dangerous circumstances, such as driving a car or operating machinery while intoxicated, is common. Furthermore, individuals with AUD may persist in drinking despite being aware of the significant physical, psychological, social, or interpersonal problems that result (4).

The framework outlined by Scafato (1) in the Report of the Italian National Institute of Health highlights the urgency of considering “harmful consumers” of alcohol as subjects “in need for treatment” pursuant to the DSM-5, clinically assimilating them to alcohol dependents. This perspective underlines the need for timely management and intervention in local services.

In a bio-psycho-social perspective, Sliedrecht and colleagues (7), identified several factors associated with AUD relapse, including the presence of psychiatric comorbidity, duration of abstinence, substance use disorder (polyaddiction), smoking, age (particularly the age of onset of AUD), sex, family history of AUD, physical health status, employment status, socioeconomic status, education level, presence of a supportive relationship (such as marriage), and motherhood for women. These factors contribute to a deeper understanding of the disorder and its involved variables.

Several psychological dimensions play a direct role in the achievement and maintenance of alcohol abstention. The interconnection between AUD and depression is well-documented, with numerous studies revealing close and complex links between the two disorders. Research by Boden and Fergusson (8) indicates that AUD significantly increases the risk of developing depression, which can manifest bidirectionally or coexist in comorbidity. Sadock et al. (9)highlight that while 80% of individuals with AUD report intense negative affect, only about 10% meet criteria for an independent diagnosis of depression. Treatment for AUD often leads to rapid improvement in depressive symptoms (10–14). Recent research by Hallgren et al. (15) suggests that reduction in alcohol consumption is associated with decreased prevalence of positive depression screenings. However, depression comorbid with AUD predicts less favorable treatment outcomes (16, 17). Similarly, heavy alcohol users have an increased risk of depression even after reducing alcohol consumption (18). Depression comorbid with AUD is associated with poorer clinical outcomes, including higher rates of treatment dropout, relapse risk, and rapid post-treatment relapse (19–21). Depression and hopelessness are closely intertwined, with research consistently indicating an increased risk of suicide among individuals dealing with AUD (22–28). Hopelessness, closely linked to suicidal intent, is prevalent in individuals with AUD further increasing suicide risk (29–31). Research indicates that alcohol can be used as a form of self-medication to manage dysphoric affects, suggesting that hopelessness may exacerbate alcohol use or other impulsive behaviors (32–34). Regarding AUD and impulsivity, studies highlight impulsivity’s significant contribution to AUD severity (35–38). High impulsivity levels in AUD patients are associated with family history of AUD and characterize individuals who drink to experience pleasurable effects (39, 40). Impulsivity, known to predict relapse during treatment, particularly early relapse, significantly impacts post-treatment quality of life and well-being. This tendency toward impulsive behaviors not only jeopardizes the effectiveness of treatment interventions but also undermines efforts to maintain long-term sobriety (7, 41–44). Interestingly, impulsivity’s adverse effects on treatment outcomes may be compounded by its association with alexithymia (45). Research indicates that alexithymia serves as a significant risk factor for the development and severity of AUD, with alexithymia individuals exhibiting a propensity to consume more alcohol and experiencing poorer treatment outcomes (46–48). This suggests a potential synergistic relationship between impulsivity and alexithymia, wherein impulsivity exacerbates emotional dysregulation, while alexithymia amplifies impulsive behaviors, creating a challenging cycle that impedes successful recovery from AUD (49–56).From the study of the literature, we can grasp the importance of abstinence as a goal for the treatment of patients with AUD. Possible variations overtime of the impact of psycopathological predictors on the risk of relapse are still unclear. Evidence suggests that certain personality traits and impulsivity play a more important role than other factors in early phases of addiction treatment, whereas the effect of alexithymia on AUD seems to be mediated by negative mood and craving, which might be sustained over time (47). Despite the large quantity of studies on the positive effects of prolonged abstinence, there is a scarcity of studies investigating the possible presence of modifications or advantages of abstinence in relation to psychological dimensions such as depression, hopelessness, impulsivity, and alexithymia in a population of patients with AUD. Given the high influence of these dimensions on the quality of life and more generally on the treatment outcomes of patients with AUD, we decided to conduct a study with the aim to verify whether there were substantial differences with respect to these dimensions between abstinent and active drinkers diagnosed with AUD.

## 2 Methodology

### 2.1 Participants

We enrolled 150 outpatients (100 males, 50 females) with a diagnosis of AUD according to the DSM-5 (57).The patients were recruited from the outpatient alcohol unit at the Ligurian Regional Alcohol Centre (San Martino Hospital, Genoa). The study was approved by the Institutional Review Board and national regulatory authorities in accordance with local requirements and was conducted in accordance with Good Clinical Practice Guidelines and the Declaration of Helsinki (1964) and subsequent revisions (D.3.2.764.14). After receiving information on the intervention, all subjects provided written informed consent.

Once the reference population was identified (patients who regularly attend the department for detoxification and/or check-ups N=245), the appropriate sample size was calculated for the analyses to be carried out using the online program “Sample Size Calculator” of the Creative Research Systems provided by the Istituto Superiore della Sanità (58). The analyses showed that 150 subject measurements were necessary to have a confidence level of 95%, therefore it was decided that the sample size should be n=150 patients using simple random extraction.

The inclusion criteria chosen to understand if a patient was eligible to enrol were: having a diagnosis of AUD according to DSM-5, being adults (aged 18 and over), having mastered the spoken and written Italian language, not being in a state of acute alcohol or drug intoxication at the time of the test and the absence of organic brain syndromes (e.g. Wernicke – Korsakoff syndrome or alcoholic encephalopathy).

At the time of testing, 78 outpatients were active drinkers while 72 outpatients were abstinent (we considered a patient abstinent after 12 weeks of sobriety, the mean time of abstinent group was 64 weeks).

### 2.2 Measurements of depression, hopelessness, impulsivity and alexithymia

Data of sociodemographic and clinical interest were collected through a specifically constructed questionnaire. Furthermore, 4 psychometric scales were administered: Beck Depression Inventory II (BDI-II), Beck Hopelessness Scale (BHS), Barratt Impulsiveness Scale (BIS-11), Toronto Alexithymia Scale (TAS-20). The BDI developed by Beck and colleagues in 1961 is one of the most used self-report questionnaires for the self-assessment of depression in clinical and research settings. This scale is made up of 21 items that allow us to detect the behavioral manifestations of depression and measure their level of severity. BHS (59)is one of the first instruments used in a clinical setting to measure patients’ suicidal risk. The BHS is a self-report questionnaire consisting of 20 items assessed on a dichotomous response scale (True or False). For the total scoring of the scale, a score of 1 or 0 is assigned depending on whether the subject answered true or false to an item and whether the item is formulated in a positive or negative key. (BIS) is a standard instrument that has had an influence in shaping current theories of impulse control and has played a key role in studies of impulsivity and its biological, psychological, and behavioral correlates (60).

BIS is made up of 30 items to which the subject responds on a Likert-type scale ranging from 1 (never/rarely) to 4 (almost always/always), the final score is therefore given by the sum of the item scores. TAS-20 (61) is one of the most used instruments in the clinical setting to measure alexithymia. The TAS-20 is a self-report questionnaire composed of 20 items assessed on a 5-point Likert scale (1 = strongly disagree; 5 = strongly agree) which measures the degree of alexithymia present in the person.

### 2.3 Data analysis

The IBM SPSS Statistics program was used to carry out the data analysis (62).

Chi squares were used for nominal values, while the Mann-Whitney U test was used for differences in test scores between the drinking and abstinent groups (after checking for normality using Shapiro-Wilks). To correct the p values with multiple comparison, False Discovery Rate (FDR) was used.

## 3 Results

A total of 150 patients diagnosed with AUD were included in the study, consisting of 100 men (66.7%) and 50 women (33.3%), with a mean age of 54.10 years (SD = 11.3). Sociodemographic and clinical characteristics are summarized in **Table 1**. The sample consisted of two groups: 78 active drinkers and 72 abstinent patients. There were no statistically significant differences between active drinkers and abstinent individuals in terms of age, sex, nicotine use, annual income, or AUD family history.

**Table 1:** Descriptives of the sample comparing abstainers and active drinkers.

	*Total sample*	*Active Drinkers*	*Abstainers*	*p value*
*Age*	54.10 ± 11.30	53.00 ± 11.55	55.29 ± 10.98	.216
*Sex (male)*	100	54	46	.448
*AUD_familiarity*	63	27	36	.056
*Income >15K*	57	26	31	.220
*Nicotine users*	95	50	45	.839
*Abstinence mean*	–	–	64 weeks	–

**Figure 1:**
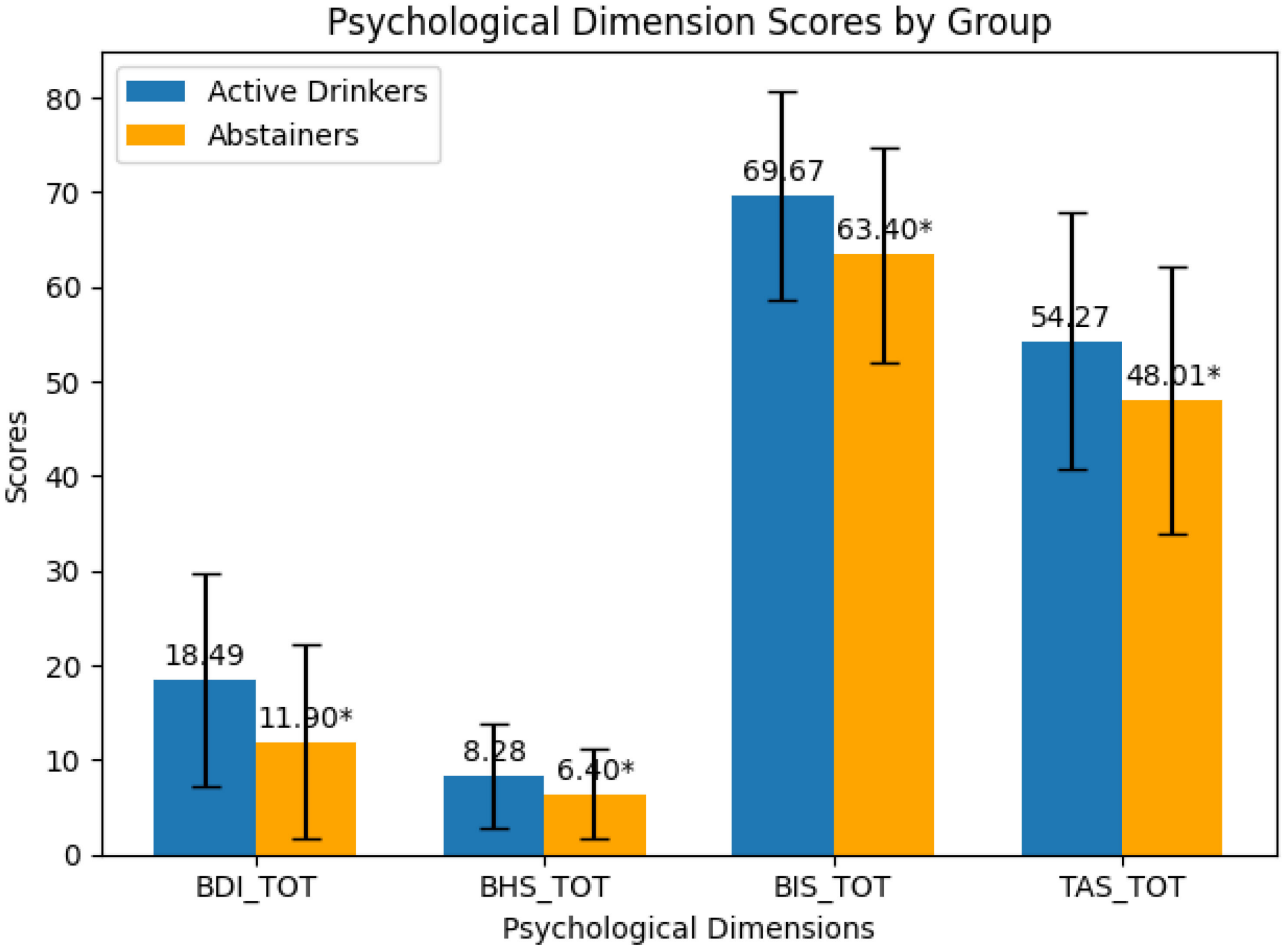
Graphical score of psychological dimensions.

Results from the non-parametric analysis corrected for multiple comparisons (FDR) are shown in **Table 2** and **Figure 1**. Active drinkers had significantly higher BDI-II scores compared to abstinent patients (Mann-Whitney U = .0001, p = .004). The mean BDI-II score for active drinkers was 18.49 (SD = 11.31) while for abstinent patients it was 11.90 (SD = 10.28). BHS scores were higher in active drinkers than in abstinent patients (Mann-Whitney U = .030, p = .030). Active drinkers had a mean BHS score of 8.28 (SD = 5.50), whereas abstinent patients had a mean score of 6.40 (SD = 4.78). According to the BIS-11, active drinkers exhibited higher impulsivity levels compared to abstinent patients (Mann-Whitney U = .001, p = .002). The mean BIS-11 score for active drinkers was 69.67 (SD = 11.09) versus 63.40 (SD = 11.29) for abstinent patients. TAS-20 scores indicated that active drinkers had higher levels of alexithymia than abstinent patients (Mann-Whitney U = .012, p = .016). The mean TAS-20 score was 54.27 (SD = 13.56) for active drinkers, compared to 48.01 (SD = 14.20) for abstinent patients.

**Table 2:** Psychological dimensions scores in abstinent vs active drinkers.

	Total Sample		Active Drinkers		Abstainers		Mann-Whitney	
*Variable*	*Mean*	*St Dev*	*Mean*	*St Dev*	*Mean*	*St Dev*	*p value*	*p value* *corrected (FDR)*
*BDI_TOT*	15.327	11.284	18.487	11.307	11.902	10.280	**.0001**	**.004**
*BHS_TOT*	7.380	5.236	8.282	5.498	6.402	4.784	**.030**	**.030**
*BIS_TOT*	51.267	14.176	69.667	11.094	63.402	11.292	**.001**	**.002**
*TAS_TOT*	66.660	11.586	54.269	13.562	48.013	14.200	**.012**	**.016**

BDI_TOT, Beck Depression Inventory score; BHS_TOT, Beck Hopeless Scale score; BIS_TOT, Barratt Impulsiveness Scale score; TAS_TOT, Toronto Alexithymia Scale score. Bold means statistically significant values (p< .05).

Rating results showing significant and non-significant comparisons in abstinent vs active drinkers. Asterisks represent significant comparisons (p<.05). BDI= Beck Depression Inventory - BHS= Beck Hopelessness Scale - BIS-11= Barratt Impulsiveness Scale - TAS20= Toronto Alexithymia Scale.

## 4 Discussion

The results of the present study revealed significant differences in the scores of depression, hopelessness, impulsivity, and alexithymia among AUD patients, categorized as either active drinker or abstinent. These findings are in line with previous studies and offer important insights into understanding the complexities of AUD and its implications for physical and mental health. It emerged that participants who continued to drink showed significantly higher levels of depression compared to their abstinent counterparts. This finding aligns with previous studies establishing a close association between alcohol consumption and depression (8). Excessive alcohol use may act as a coping mechanism for dealing with depressive symptoms; however, depression itself may also increase the risk of AUD, creating a vicious cycle that warrants particular attention in clinical practice. During withdrawal from alcohol, there is a focus on reduced experiences of reward and increases in negative affect, which can then contribute to the restart of alcohol consumption. These psychological experiences are coupled with changes in the striatum, extended amygdala, and insula functioning. These brain structures are critical nodes involved in the salience network (SN) (63). SN is highly relevant to the development and maintenance of AUD by influencing negative affect (64), incentive salience (65), and executive function (66) networks. Reduction in alcohol consumption is associated with a decrease in the prevalence of positive screening for depression (15) and mood-related symptoms tend to dissipate within the first weeks for the majority of patients (11). The results also indicated that active-drinker participants reported higher impulsivity scores compared to abstinent patients. Impulsivity has been extensively studied in the context of AUD and has proven to be a significant risk factor for its development and maintenance (35–38). Impulsivity can influence the ability to resist the temptation to drink and may also contribute to a lack of awareness of the risks associated with AUD (39). Subsequentially, active-drinker reported higher levels of alexithymia compared to abstinent patients. Alexithymia, the difficulty in identifying and expressing emotions, has been associated with increased vulnerability to the negative effects of substance misuse (52, 53) and previous studies found that alexithymic individuals appear to consume greater quantities of alcohol than non-alexithymic individuals (51).Alcohol’s numbing effect on emotions can hinder individuals’ ability to identify and express their feelings, leading to interpersonal difficulties and psychological distress. Additionally, the presence of alexithymia may further complicate the treatment of AUD as a lack of emotional awareness can impede effective participation in therapy and recovery programs. Finally, the higher levels of hopelessness reported by active-drinkersraise significant concerns about the mental health and well-being of those struggling with AUD. The association between AUD and suicide has been well-documented and demands immediate attention from mental health professionals to enact effective prevention strategies and targeted interventions (24, 26, 27, 67). The correlation between alcohol use and suicidal ideation highlights the life-threatening consequences of AUD. Alcohol’s depressant effects can exacerbate feelings of hopelessness and despair, increasing the likelihood of suicidal thoughts and behaviors. As these dimensions are known risk factors in predicting alcohol relapse in a time-varying manner (42), our results emphasize the importance of recognizing and treating the different degrees of severity of these dimensions from a personalized care perspective.

The investigation of psychopathological dimensions, may be useful to identify different phenotypes of AUD patients. The baseline assessment of specific features, that is, impulsivity, hopelessness depression and alexithymia, among others, could identify patients more at risk for relapse and support clinicians to adjust therapeutic program during crucial moments of treatment and follow up. This implies that, maintaining low levels of impulsivity, alexithymia, depression and hopelessness are of primordial importance for relapse prevention. This can be done through the choice of the right integrated therapy with regard to the specific patient. The choice of appropriate therapy can also benefit from the use of digital assessment methods. Among the various tools available to the clinician to monitor a patient’s daily life and response mechanisms to stressors, we can mention the ecological momentary assessment (EMA). EMA describes a type of data collection that allows clinicians and researchers to gather detailed insight into the daily lives of patients by inquiring about the subjects’ mental state in the moment, avoiding memory recall bias. EMA techniques provide methods by which a patient can report on symptoms, affect, behavior and cognitions close in time to experience, and these reports are obtained many times over the course of a day (68).

Psychological treatment of AUD is of considerable importance to improve patients’ quality of life, avoiding clinical treatments that lead to discontinuation. Approaches based on motivational interviewing and cognitive behavioral therapy (CBT) are the most effective and appropriate psychological treatments for AUD (69). At the same time, it has been shown that the 12 Steps program (70)appears to be very effective and produces economic benefits (71), (72), (73)Mindfulness-Based Therapies have also shown promise in treating AUD by helping patients increase their awareness and acceptance of cravings without acting on them, thus reducing relapse rates. Furthermore, Interpersonal Therapy (IPT) focuses on improving interpersonal functioning and has been found to be beneficial in treating AUD by addressing the social and relational issues that often accompany the disorder (74, 75). On the other side, pharmacological treatments for AUD may include different approaches to address various stages and symptoms of this condition. Disulfiram is used for alcohol aversion, it induces unpleasant reactions when drinking alcohol, such as nausea, vomiting and headaches. This helps to dissuade the patient from drinking. Methadoxine works by reducing alcohol levels in the blood and facilitating its elimination in the urine. Acamprosate leads to a reduction of glutamatergic excitability during alcohol withdrawal reducing the risk of alcohol consumption and to increase the total duration of abstinence (76). Naltrexone blocks the euphoric effects associated with alcohol (77). Benzodiazepines can be used to manage withdrawal and reduce anxiety. The different mechanisms of action and effects of these drug therapies, in conjunction with brief weekly adherence counseling, rehabilitation or psychotherapy, can only give their best when paired with the specific psychopathological constructs of the patient (78). This means that to target alcohol abstention, instead of only turning the patient away from the substance use, we will need to better understand his or her specific dimensions and treat that in the first place.

Some limitations must be acknowledged like the cross-sectional design of our study, which involves observing participants at a single point in time. This approach limits our ability to establish cause-and-effect relationships between the variables examined. Longitudinal studies may be necessary to evaluate dynamics over time. Also, it should be noted that the sample size, although appreciable is limited to 150 subjects. This restriction could affect the generalizability of the findings to a broader population. Further studies with larger samplings are needed to confirm and generalize our findings.

Moreover, we did not consider the presence of other comorbidities such as ADHD and personality disorders, which could influence the results of our study. The exclusion of these variables may limit the comprehensiveness of the analysis.

Finally, the use of self-administered questionnaires can be subject to self-report bias and variability in participants’ understanding and interpretation of the questions, thus affecting the accuracy of the data collected.

Conversely, the study has the following strengths: the real-world outpatient setting, the naturalistic design and the comprehensive assessment of the four psychopathological constructs.

## 5 Conclusion

In conclusion, the results of this research confirm the importance of understanding the multifaceted nature of the AUD. One of the key implications of this research is the critical role played by specific psychopathological dimensions in alcohol abstinence. By abstaining from alcohol or moderating its use, individuals can mitigate the risk of developing or worsen the course of various mental health conditions, promoting a positive circle. The relationship between alcohol abstinence and mental health is reciprocal: prioritizing mental well-being can support efforts toward alcohol abstinence, while abstaining from alcohol can contribute to improved mental health outcomes.

Future studies, should use a longitudinal approach and with the help of digital assessment methods, identify the different stages of AUD in relation to the different psychopathological constructs and consequently the different specific lines of treatment.

## Data availability statement

The raw data supporting the conclusions of this article will be made available by the authors, without undue reservation.

## Ethics statement

The studies involving humans were approved by Comitato Etico per la Ricerca di Ateneo (CERA). The studies were conducted in accordance with the local legislation and institutional requirements. The participants provided their written informed consent to participate in this study. Written informed consent was obtained from the individual(s) for the publication of any potentially identifiable images or data included in this article.

## Author contributions

AZ: Writing – review & editing, Writing – original draft, Data curation. IB: Writing – review & editing, Writing – original draft, Investigation. AB: Writing – review & editing, Writing – original draft, Conceptualization. MO: Writing – review & editing, Writing – original draft, Data curation. AE: Writing – review & editing, Methodology. RG: Writing – review & editing, Visualization. BD: Writing – review & editing, Software. MA: Writing – review & editing, Supervision. GS: Writing – review & editing, Supervision.
